# Edge-Cloud Synergy for AI-Enhanced Sensor Network Data: A Real-Time Predictive Maintenance Framework

**DOI:** 10.3390/s24247918

**Published:** 2024-12-11

**Authors:** Kaushik Sathupadi, Sandesh Achar, Shinoy Vengaramkode Bhaskaran, Nuruzzaman Faruqui, M. Abdullah-Al-Wadud, Jia Uddin

**Affiliations:** 1 Google LLC, Sunnyvale, CA 94089, USA; sathupadi.kaushik@gmail.com; 2 Department of Software Engineering, Walmart Global Tech, Sunnyvale, CA 94086, USA; sandeshachar26@gmail.com; 3 Zoom Video Communications, Sanjose, CA 95113, USA; shenoyvb@gmail.com; 4 Department of Software Engineering, Daffodil International University, Daffodil Smart City, Birulia, Savar, Dhaka 1216, Bangladesh; faruqui.swe@diu.edu.bd; 5 Department of Software Engineering, College of Computer and Information Sciences, King Saud University, Riyadh 11543, Saudi Arabia; 6 AI and Big Data Department, Endicott College, Woosong University, Daejeon 34606, Republic of Korea

**Keywords:** sensor network, hybrid edge-cloud framework, predictive maintenance, K-nearest neighbors (KNN), long short-term memory (LSTM) network, sensor networks, latency optimization, energy efficiency, bandwidth reduction, dynamic workload management

## Abstract

Sensor networks generate vast amounts of data in real-time, which challenges existing predictive maintenance frameworks due to high latency, energy consumption, and bandwidth requirements. This research addresses these limitations by proposing an edge-cloud hybrid framework, leveraging edge devices for immediate anomaly detection and cloud servers for in-depth failure prediction. A K-Nearest Neighbors (KNNs) model is deployed on edge devices to detect anomalies in real-time, reducing the need for continuous data transfer to the cloud. Meanwhile, a Long Short-Term Memory (LSTM) model in the cloud analyzes time-series data for predictive failure analysis, enhancing maintenance scheduling and operational efficiency. The framework’s dynamic workload management algorithm optimizes task distribution between edge and cloud resources, balancing latency, bandwidth usage, and energy consumption. Experimental results show that the hybrid approach achieves a 35% reduction in latency, a 28% decrease in energy consumption, and a 60% reduction in bandwidth usage compared to cloud-only solutions. This framework offers a scalable, efficient solution for real-time predictive maintenance, making it highly applicable to resource-constrained, data-intensive environments.

## 1. Introduction

### 1.1. Background and Motivation

The rapid adaptation of sensor networks across various industries has made real-time monitoring, automation, and data-driven decision-making possible. Smart cities’ emergence and expeditious expansion are also the blessings of sensor networks [1]. However, real-time sensor network management is challenging because of the 3 Vs of Big data: volume, velocity, and variety [2]. Preprocessing the data with edge computers is a potential solution to overcome the challenge. It also reduces the latency and bandwidth requirements for data transmission. However, the computational limitations of the cost-effective edge devices curve the scope of utilizing Deep Learning (DL) models [3]. On the other hand, cloud computing offers powerful resources for large-scale data processing but suffers from network latency and potential bandwidth bottlenecks [4]. This dichotomy between cloud and edge computing has created the need for a synergistic framework where edge computers use lightweight models, and heavy computing is delegated to the heavier models hosted in the cloud servers. This hybrid approach set the foundation for predictive maintenance that enhances the operational efficiency of sensor networks, ensuring timely maintenance interventions and reducing downtime [5]. As a result, the predictive maintenance framework effectively optimizes the repair schedules, minimizes disruptions, and extends the lifespan of equipment.

### 1.2. Problem Statement

Significant advancements have been made in sensor network-based predictive maintenance systems. However, the existing field of research is dominated by reliance on either edge computing or cloud servers [6]. This bifurcation limits their efficiency in handling real-time sensor data streams effectively. On the one hand, edge computers are efficient in low-latency responses. However, their computational capability is unsuitable for complex Artificial Intelligence (AI) models [7]. On the other hand, cloud computing provides extensive computational power. However, transferring the big data introduces latency and bandwidth challenges. In addition, the existing predictive maintenance systems struggle with dynamic workload issues caused by sensor data volume fluctuations [8]. As a result, a rigid reliance on either edge or cloud resources can result in bottlenecks, leading to delayed responses and missed predictions. Ensuring optimal resource utilization is another critical challenge in cloud computing. Inefficient use of computational and network resources can lead to excessive energy consumption and increased operational costs [9]. As a result, it impacts the feasibility of the predictive maintenance frameworks in sensor networks in real-world, real-time applications. A seamless synergy between edge and cloud is required to balance these trade-offs while ensuring continuous sensor operations and accurate maintenance predictions. That is why a hybrid edge-cloud framework is needed to enable real-time decision-making for predictive maintenance systems. The hybrid approach solves the bottlenecks of either an edge or cloud-based approach. However, the problem is to ensure that the framework performs continuous data processing, minimizes energy consumption, and dynamically allocates workloads based on network conditions to prevent operational bottlenecks.

### 1.3. Research Objectives

Design, implementation, and experimentation with a hybrid edge-cloud framework for AI-enhanced sensor networks with the capability of real-time predictive maintenance that ensures optimal cloud resource consumption is the primary objective of this study. The aim of the framework is to utilize both edge and cloud computing to achieve a balanced trade-off among computing resource use, network latency, and energy consumption. The specific objectives of the research are as follows:

- Hybrid AI Model Deployment: Developing a lightweight AI model suitable for edge devices for real-time anomaly detection complemented by deep learning models in the cloud for comprehensive analytics.
- Dynamic Workload Management: Designing a novel algorithm to dynamically balance workloads between edge devices and cloud servers depending on network conditions, data volume, and sensor operations while ensuring uninterrupted performance.
- Predictive Maintenance: To implement a full-fledged predictive maintenance system that analyzes time-series sensor data, predicts potential equipment failures and optimizes maintenance schedules to facilitate improved operational efficiency.
- Resource Optimization: Evaluate strategies for efficient resource allocation between the edge and cloud to minimize energy consumption, network bandwidth usage, and latency.

Overall, this paper aims to utilize the advantages of both edge devices and cloud computing by coupling them through a novel real-time predictive maintenance framework that efficiently balances complementary characteristics of both systems while optimizing resource consumption.

### 1.4. Core Contributions

This paper contributes to the application domain by conducting rigorous studies on edge-cloud synergy. Applying existing AI solutions in under-explored domains introduces a novel solution to the edge-cloud bottleneck problem in sensor networks for real-time predictive maintenance systems. Coining a hybrid AI model deployment methodology equipped with an innovative, dynamic workload management algorithm for time-series data-dependent predictive maintenance to optimize resource utilization, ensuring energy and bandwidth, significantly contributes to sensor network research. The core contributions of this research are outlined as follows:

- Hybrid AI Model Deployment: We propose the development of lightweight AI models deployed on edge devices to perform immediate anomaly detection. These models operate in conjunction with cloud-based deep learning algorithms, which provide comprehensive analytics and long-term insights.
- Dynamic Workload Management Algorithm: A novel algorithm for real-time workload offloading is introduced, which dynamically shifts workloads between the edge and cloud-based on sensor activity, network conditions, and computational demands. This ensures seamless operation and prevents bottlenecks.
- Predictive Maintenance Using Time-Series Data: The framework utilizes AI to analyze sensor data streams, predicting potential equipment failures. By doing so, it enables proactive scheduling of maintenance, reducing downtime and extending equipment lifespan.
- Resource Optimization for Energy and Bandwidth Efficiency: We investigate resource allocation strategies to optimize the distribution of computational tasks between the edge and cloud. The goal is to minimize energy consumption and bandwidth usage while maintaining low-latency operations.

The remaining parts of this paper have been organized into six different sections. The relevant literature review is presented in Section 2, which supports the objectives of this paper. Section 3 presents the methodological development. The implementation of the proposed system is in Section 4. Section 5 presents the experimental results and evaluates the overall effectiveness of the system. The limitations of the proposed system are highlighted in Section 6. Finally, the paper has been concluded in Section 7.

## 2. Literature Review

### 2.1. Edge Computing in AI-Enhanced Sensor Networks

According to the study conducted by Sharma et al. [10], edge computing reduces latency and facilitates real-time decision-making. As a result, it is becoming increasingly relevant to sensor networks. A literature review conducted by Wu et al. [11] shows that the integration of AI algorithms to edge computers in sensor networks has gained widespread popularity for anomaly detection, data filtering, and initial processing. However, only lightweight AI models are suitable for edge devices because of the limited resource capability [12]. Techniques such as model pruning, quantization, and knowledge distillation have been applied to make AI models more suitable for edge deployment [13]. While these approaches enable edge devices to execute basic AI functionalities, they are not enough for complex analytics. The rigorous survey concluded that edge computers are effective for low-latency response. However, it struggles with constraints on computational power, memory, and energy efficiency [14]. This is the main barrier to implementing deep learning models in these devices for advanced analytics, as they require a significant amount of computing resources [15]. This paper aims to contribute to this research domain by presenting a hybrid approach that combines the strength of both edge and cloud computing to enhance the performance of sensor networks.

### 2.2. Cloud-Based AI Analytics

It is no exaggeration to say that cloud computing offers unlimited computing resources [16]. According to the methodology of [17,18,19], cloud-based platforms are feasible for hosting a complex DL model to develop real-time predictive analytics. Even if the model requires high-capacity storage and processing power, cloud servers are capable of allocating them. Concentrating all data in the cloud, training the DL model on those, and applying the model to develop advanced analytics from multiple sensors can provide insights beyond the capabilities of individual edge devices, helping to identify patterns and correlations across sensor data streams [20]. According to Ren et al. [21], a sensor network produces big data, and transmitting those to the cloud introduces significant network latency. As a result, real-time decision-making suffers from delays, often compromising the quality of services. The study conducted by [22] discusses the bandwidth cost and network resource consumption of big data transmission. According to this study, these costs grow rapidly to an unsustainable level unless appropriately managed. These issues highlight the need for a balanced approach, where the cloud’s analytical strengths are combined with the immediacy of edge processing to optimize predictive maintenance in sensor networks.

### 2.3. Hybrid Edge-Cloud Frameworks

Hybridization, which combines edge devices and cloud computing to leverage the complementary advantages of these two technologies, is a popular field of research. Multiple advancements have already been made in this area [23]. Grzesik et al. [24] conducted a rigorous study on frameworks that combine edge computing with color servers and summarized the opportunities and challenges. A similar study was conducted in [25] as well. These researches point toward the under-explored solutions to the challenges of managing dynamic workloads in sensor networks, which require intelligent decision-making to prevent bottlenecks and ensure resource efficiency. Moreover, the need for balanced energy consumption and bandwidth optimization are two marginally explored research areas. These gaps underscore the need for optimized workload management algorithms and resource allocation strategies to fully realize the potential of hybrid edge-cloud frameworks.

### 2.4. Predictive Maintenance in Sensor Networks

Predictive maintenance is emerging as a popular approach in industrial production, smart solutions, and automation because of its potential to minimize downtime and optimize maintenance schedules [26]. The underlying technology is the utilization of time-series data from the sensor networks in combination with AI models to forecast when maintenance is needed [27]. According to Maguluri et al. [28], AI-based predictive maintenance relies on Machine Learning (ML) and Deep Learning (DL) to identify trends, anomalies, and failure precursors within the data streams. Time-series data analysis plays a crucial role here where Recurrent Neural Networks (RNNs) and Long Short-Term Memory (LSTM) models are frequently used [29]. This is where the main challenge arises, as the complexities of these models are too high to run effectively on edge devices. Moreover, the frequency and variety of the data produced by the sensor networks overwhelm the edge and cloud resources. It leads to latency issues, and the systems start consuming excessive energy [30]. However, the state-of-the-art studies [31] and traditional predictive maintenance framework [32] exhibit a lack of mechanisms to balance real-time processing demands with resource efficiency. This calls for the development of hybrid approaches that can leverage edge processing for immediate anomaly detection and cloud analytics for in-depth failure prediction, thus maximizing both accuracy and efficiency.

### 2.5. Recent Advancements in Hybrid Edge-Cloud Frameworks

A rigorous review conducted by Boiko et al. [33] reveals the recent advancement in Hybrid Edge-Cloud frameworks. KRIOTA [34] is one of these frameworks, which is a situation-aware system for robotics. Although it performs well for predefined environments, unlike the proposed framework, it lacks adaptability to various environments. The ArchGPT developed by [35] comes with dynamic and adaptive algorithms to optimize resource allocation and task distribution. However, it does not explicitly incorporate the sensor network like the proposed framework does. An intelligent water quality monitoring system developed by Shahra et al. [36] is another recent application of edge-cloud hybridization. Bhoi et al. [37] applied the concept of edge-cloud hybridization in power monitoring in electronics systems. However, in these studies, the combination of dynamic workload management, predictive maintenance, and resource optimization is missing as the proposed methodology. It makes the approach presented in this paper the most comprehensive and practical approach, which is adaptable to various applications and capable of maintaining scalability within a wide range of data ingestion variations.

## 3. Methodology

### 3.1. Dataset Description and Preprocessing

This study uses a dataset consisting of historical time-series data collected from multiple sensors in an industrial setting. This dataset has been used to train the KNN model for anomaly detection. The LSTM learned to predict sensor failure from the same dataset. Key features include time, sensor readings, operational status, and failure labels.

#### 3.1.1. Dataset Features

The dataset used in this paper contains mainly time-series features. These features are related to anomaly detection and failure prediction, which are recorded on the dataset. Each record contains a Timestamp *t* that specifies the precise time of the reading. This dataset contains the data of 20 sensors, which have been modeled as $X_{t}=\left\{x_{t1},x_{t2},\ldots ,x_{t20}\right\}$. Each sensor $x_{ti}$ is the measurement from the *i*-th sensor at time *t*. A sample of the dataset is presented in Table 1 where up to six sensor readings are shown. In this dataset, $y_{t}$ is a binary label indicating the normal or anomalous state of operation, which is used to train the KNN model for real-time anomaly detection. The last column of the dataset contains the Failure States $z_{t}$ features. The LSTM network used in this paper has been trained considering $z_{t}$ as the target variable.

#### 3.1.2. Data Preprocessing

The data from the sensors come in normalized form with a range from 0 to 1. As a result, normalization is not performed during preprocessing. However, there are multiple missing values in the dataset, which require cleaning. Sequential segmentation is also necessary for the LSTM network. Initially, the dataset has been analyzed manually using Microsoft Excel. It was observed in the initial analysis that there were missing values. Outliers have also been identified among the data. For consistent analysis, missing values were interpolated linearly, and outliers were removed. Outliers were identified by analyzing each sensor’s standard deviation and setting threshold limits to $x_{ti}$, which has been measured using Equation (1).

(1) $$x_{ti}\;\mathrm{is}\;\mathrm{an}\;\mathrm{outlier}\;\mathrm{if}\;\left|x_{ti}-\mu_{i}\right|>\alpha \cdot \sigma_{i},$$

In Equation (1), $\mu_{i}$ and $\sigma_{i}$ denote the mean and standard deviation of sensor *i*, and α is an outlier threshold factor. To train the LSTM network, the data were segmented into sequences to capture temporal dependencies. Each input sequence $X_{t}$ spans *n* time steps, as expressed in Equation (2), where $X_{t-i}$ represents the feature vector at time t−i. The *n* is the sequence length. The target label for each sequence is the failure status $z_{t+k}$ after a prediction horizon *k*.

(2) $$X_{t}=\left\{X_{t-n+1},X_{t-n+2},\ldots ,X_{t}\right\},$$

There are a total of 123,013 instances in the dataset. It has been split into training, testing, and validation datasets with a ratio of 70:15:15. A temporal split was performed to maintain the chronological order of the data. The splitting supports the union operation, as defined in Equation (3) [38].

(3) $$D=D_{\mathrm{train}}\cup D_{\mathrm{val}}\cup D_{\mathrm{test}},$$

### 3.2. Comparative Analysis for Hardware and AI Model Selection

The development of the predictive maintenance framework starts with selecting appropriate hardware and AI models. This has been achieved through comparative analysis. Computational capability, power consumption rate, cost, computational complexity, and accuracy are the selection criteria used in this section.

#### 3.2.1. Edge Device Selection

The network sensors are connected to the edge devices, which perform the initial processing. The more sensors there are, the more devices will be required. Massive sensor networks require a lot of edge devices. That is why going for cost-effective devices is always preferred. However, making the right decision based on the trade-off between computational capability and cost is challenging. Table 2 has been prepared to address this challenge.

According to the comparative analysis in Table 2, the Raspberry Pi Zero 2W offers a balanced trade-off between processing power and power consumption. This trade-off makes it suitable for real-time anomaly detection in resource-constrained environments. Moreover, its low cost and moderate CPU performance support the deployment of lightweight AI models enough to detect the initial anomaly. That is why the Raspberry Pi Zero 2 W has been used as the edge device in this study.

#### 3.2.2. AI and ML Model Selection

The AI model for the edge device must be lightweight and computationally efficient. As the edge devices are resource-constrained environments, complex models that require extensive memory are not feasible. From this perspective, a comparative study has been performed, and the outcomes are listed in Table 3. Based on the feature comparison of Table 3, the KNN has been selected as the AI model of the edge computer [39].

Although Table 3 shows that it has a moderate level of accuracy, it has been selected because of its low computational complexity and memory usage, making it suitable for deployment on the Raspberry Pi Zero 2 W. Although Decision Tree and SVM models provide higher accuracy, they are more resource-intensive, which would strain the edge device’s limited resources. Among the compared models, only KNN offers a reliable balance among computational complexity, memory usage, and accuracy. Considering the trade-off and practicality of the system, this research continued with KNN as the finalized AI model for edge devices.

#### 3.2.3. Cloud Service and Model Selection

The experimental phase of this research requires the capability of handling massive volumes of data with numerous varieties at a very high velocity. That is why the primary selection criteria of the cloud service provider are high scalability and low latency. After the successful implementation of the proposed framework, the cloud server cost will be reduced. With this vision, the cost of provisioning the cloud server was less emphasized. The findings of the comparative analysis on different cloud service providers are listed in Table 4 [40].

Although the AWS Labda is costlier than other service providers, it has been selected for this research, considering its scalability and latency parameters. Furthermore, its ability to handle dynamic workloads is advantageous for deep learning applications that require significant computational resources.

#### 3.2.4. Deep Learning Model Selection

The DL models run on the cloud, where the availability of computational resources is not an issue. That is why the suitability of time-series data analysis and accuracy have been given priority while making the selection. The findings of the exploratory analysis performed in this paper to identify the most suitable DL model are listed in Table 5.

According to multiple state-of-the-art studies, the LSTM model is most suitable for time-series data. Although the RNN is equally competitive in the race, it suffers from performance issues for long sequences. As we deal with big data, the data sequence length may vary significantly. In addition, GRU and CNN are not as promising as LSTM in terms of time-series data analysis [41,42,43,44]. Considering everything, the LSTM network has been selected to deploy in the cloud server to develop the predictive maintenance system.

#### 3.2.5. Addressing Retraining Challenges in Machine Learning Models

Retraining machine learning models is critical for maintaining their performance over time, especially in dynamic environments such as sensor networks. The proposed hybrid framework incorporates strategies to address retraining challenges, ensuring that the KNN and LSTM models remain effective despite changes in data patterns or system conditions.

To minimize the need for complete retraining, the KNN model deployed at the edge is enhanced with incremental learning capabilities. Instead of retraining the model from scratch, the system updates the nearest neighbor data incrementally as new data points are received. This approach significantly reduces the computational overhead and ensures real-time responsiveness. The incremental update is governed by Equation (4), where $X_{new}$ represents the newly added data points.

(4) $$X=X_{old}\cup X_{new}$$

For the LSTM model in the cloud, periodic retraining is scheduled to incorporate new data and adapt to evolving patterns. To mitigate the computational burden of retraining, a subset of the most recent data, combined with a representative sample of historical data, is used for retraining. This subset selection ensures that the model captures both recent trends and long-term patterns. The retraining schedule is determined dynamically based on the rate of data drift, which is calculated using the Kullback–Leibler (KL) divergence between the historical and new data distributions, as shown in Equation (5).

(5) $$D_{KL}\left(P||Q\right)=\sum_{i}P\left(i\right)\log\frac{P(i)}{Q(i)}$$

The overall retraining workflow ensures that updates occur seamlessly without interrupting real-time operations. Edge-based incremental updates for KNN occur in parallel with ongoing anomaly detection, while LSTM retraining is conducted during periods of low system activity to minimize the impact on performance. This dual approach balances real-time performance with the adaptability required in dynamic environments.

#### 3.2.6. Justification for the Choice of Metric in KNN

The choice of Euclidean distance as the metric for the KNN method in this study is based on its computational simplicity and suitability for the normalized dataset used. Since the data features are scaled to a range of 0 to 1, the Euclidean distance effectively captures the similarity between data points without being influenced by varying feature scales.

Alternative metrics, such as the Manhattan distance and Minkowski distance, were also considered. However, these metrics showed negligible improvement in classification accuracy during preliminary experiments while incurring additional computational overhead. The comparative analysis of metrics is presented in Table 6, which highlights the trade-offs in accuracy and computational efficiency.

Based on these results, Euclidean distance was chosen as it provides a balance between high classification accuracy and low computational cost, making it well-suited for deployment on resource-constrained edge devices.

#### 3.2.7. Selection of Optimal Parameters for KNN

The performance of the KNN model is influenced significantly by two parameters: the number of nearest neighbors (*k*) and the window width for time-series segmentation. To ensure optimal parameter selection, this study conducted a grid search using cross-validation on the training dataset.

**Figure 1:**
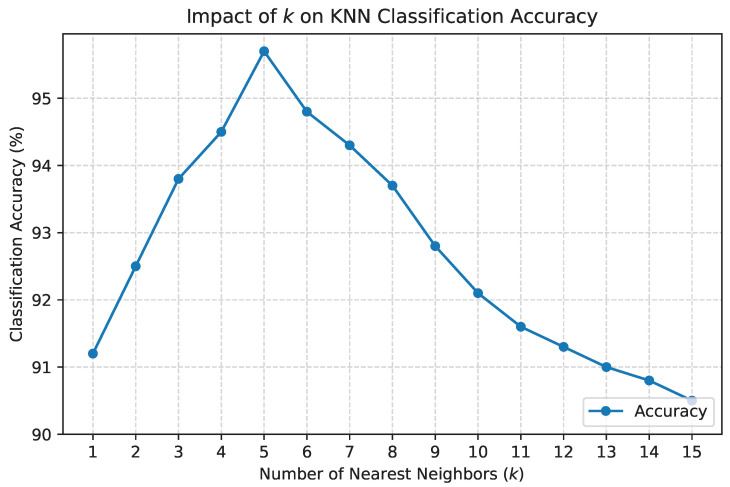
Impact of *k* on KNN classification accuracy.

The number of nearest neighbors, *k*, was varied across a range of values (k=1 to k=15) to identify the setting that maximized classification accuracy. The results of this analysis are presented in Figure 1, which shows the relationship between *k* and classification accuracy. Based on these results, k=5 was selected, as it achieved the highest accuracy while maintaining computational efficiency. For time-series segmentation, the window width was varied from 10 to 50 time steps. The optimal window width was determined by evaluating the model’s performance using a combination of metrics, including classification accuracy and latency. The results of this analysis are presented in Table 7, which shows that a window width of 30 time steps provided the best trade-off between accuracy and processing time.

### 3.3. Data Transfer Protocols and Their Limitations

Effective data transfer is critical in hybrid edge-cloud frameworks, especially for ensuring real-time communication and minimizing latency. Table 8 presents a comparison of commonly used data transfer protocols, focusing on their characteristics, limitations, and suitability for the proposed system.

### 3.4. Conceptual Design of the Hybrid AI Model

The proposed hybrid AI model combines the KNN model and the LSTM network. The KNN runs from the edge device and is designed to detect anomalies. The LSTM network operates from the cloud server to predict the failure based on the time-series data analysis. This integration enables real-time responsiveness at the edge with deeper, long-term insights from the cloud.

#### 3.4.1. Anomaly Detection at the Edge

The choice of data transfer protocols significantly impacts the performance of anomaly detection at the edge. Protocols such as HTTP introduce higher latency due to their inherent overhead, which can delay real-time detection. MQTT and WebSocket, with their low latency, are more suitable for timely anomaly alerts. However, MQTT’s reduced reliability in high-traffic scenarios may result in dropped messages. CoAP, being lightweight and efficient, is ideal for low-latency operations but struggles with scalability in larger deployments [45]. These trade-offs emphasize the importance of protocol selection in achieving consistent real-time performance.

In the conceptual design, the $x_{t}$ denotes the sensor reading received at time *t* and $X=\{x_{1},x_{2},∆,x_{n}\}$ represent a historical set of readings. The KNN model processes each new reading $x_{t}$ by computing its distance from the *k* nearest neighbors within the historical dataset *X*. After that, the anomaly score is calculated using the mathematical principle presented in Equation (6), where the anomaly score is expressed as $S(x_{t})$.

(6) $$S\left(x_{t}\right)=\frac{1}{k}\sum_{i=1}^{k}∥x_{t}-x_{\left(i\right)}∥,$$

The $x_{\left(i\right)}$ in Equation (6) represents the *i*-th nearest neighbor in *X*. The ∥·∥ represents the Euclidean distance. If $S(x_{t})$ exceeds a predefined threshold $S_{thresh}$, an anomaly is flagged, prompting a data transfer to the cloud for further analysis. The KNN model used at the edge device is computationally less complex and requires low memory requirements. As a result, it smoothly runs on the Raspberry Pi Zero 2 W.

The Euclidean distance was used as the distance metric of the KNN. It was validated through comparative experiments with alternative metrics such as Manhattan and Minkowski distances. These alternatives showed similar classification accuracy but required higher computational resources, which is a significant constraint for edge devices. The Euclidean distance was, therefore, selected as it aligns with the resource constraints of the edge devices while maintaining high accuracy. The selection of the number of nearest neighbors (*k*) and the window width for time-series segmentation significantly impacts the performance of the KNN model. Through grid search and cross-validation, k=5 and a window width of 30 time steps were determined to provide the best trade-off between classification accuracy and computational efficiency. These parameters ensured the model’s effectiveness while adhering to the constraints of the edge devices.

#### 3.4.2. Threshold Calculation for Anomaly Detection

The anomaly detection threshold $S_{thresh}$ is set based on the statistical distribution of anomaly scores $S\left(X\right)=\{S\left(x_{1}\right),S\left(x_{2}\right),\ldots ,S\left(x_{n}\right)\}$ from historical data *X*. The mean $\mu_{S}$ and standard deviation $\sigma_{S}$ of these scores are calculated using Equations (7) and (8), respectively.

(7) $$\mu_{S}=\frac{1}{n}\sum_{i=1}^{n}S\left(x_{i}\right),$$

(8) $$\sigma_{S}=\sqrt{\frac{1}{n}\sum_{i=1}^{n}{\left(S\left(x_{i}\right)-\mu_{S}\right)}^{2}}.$$

Finally, the threshold score, which is defined as $S_{thresh}$, is calculated using Equation (9). In this equation, the α is the sensitivity parameter. It is adjustable and used to control the system’s sensitivity to ensure optimal performance with a lower false positive rate.

(9) $$S_{thresh}=\mu_{S}+\alpha \cdot \sigma_{S},$$

This threshold allows the KNN model to detect meaningful anomalies, prompting cloud analysis only when necessary. That means the cloud server does not need to process each instance of the sensor. As long as the sensors are running properly, the data are not passed to the cloud server. Only when the anomaly score crosses the threshold does the sensor start sending data to the cloud server for further time-series prediction.

#### 3.4.3. Failure Prediction in the Cloud

Once an anomaly is detected, the proposed framework considers the probability of a sensor failure high. From this point, the LSTM network hosted on the cloud server starts receiving data from the sensors directly. The sensor data are time-series data and organized as $X_{t}=\left\{x_{t-1},x_{t-2},\ldots ,x_{t-n}\right\}$. The LSTM network takes $X_{t}$ as input and predicts the probability of failure within a future horizon *k*. The prediction by the LSTM network is modeled as Equation (10).

(10) $$y_{pred}\left(t+k\right)=M_{LSTM}\left(X_{t};\theta \right),$$

In Equation (10), $M_{LSTM}\left(\cdot \right)$ represents the LSTM model where the θ denotes the parameters after training. The $y_{pred}\left(t+k\right)$ represents the predicted failure probability at time t+k.

#### 3.4.4. Hybrid AI Model Operation Algorithm

The overall workflow of the proposed hybrid AI model has been presented as Algorithm 1. It demonstrates the procedure of integrating the KNN-based anomaly detection at the edge device. At the same time, it presents how the LSTM-based failure prediction in the cloud is integrated into the system. The algorithm establishes a logical connection between the components of the conceptual design, enabling efficient, real-time predictive maintenance.
**Algorithm 1** Hybrid AI Model for Predictive Maintenance1:**Input:** Sensor reading $x_{t}$ at time *t*; historical dataset $X=\{x_{1},x_{2},\ldots ,x_{n}\}$; predefined threshold $S_{thresh}$2:**Output:** Failure prediction $y_{pred}\left(t+k\right)$ for future time t+k3:**Initialize:** Load KNN model on Raspberry Pi Zero 2 W; load LSTM model on cloud server4:Compute mean $\mu_{S}$ and standard deviation $\sigma_{S}$ of historical anomaly scores S(X) using Equations (7) and (8)5:Set threshold $S_{thresh}=\mu_{S}+\alpha \cdot \sigma_{S}$ (Equation (9))6:**while** True **do**                              ▹ Continuous monitoring loop7:     Receive new sensor reading $x_{t}$ from the sensor network8:     Compute anomaly score $S(x_{t})$ using KNN (Equation (6))9:     **if** $S\left(x_{t}\right)>S_{thresh}$ **then**10:         **Flag anomaly** and send $x_{t}$ to cloud server11:         Collect recent time-series data $X_{t}=\left\{x_{t-1},x_{t-2},\ldots ,x_{t-n}\right\}$12:         Pass $X_{t}$ to LSTM model on the cloud server13:         Predict failure probability $y_{pred}\left(t+k\right)=M_{LSTM}\left(X_{t};\theta \right)$ (Equation (10))14:         **if** $y_{pred}\left(t+k\right)$ exceeds predefined failure probability threshold **then**15:             **Trigger maintenance alert** and log data for further analysis16:         **else**17:             Continue monitoring and anomaly detection at the edge18:         **end if**19:     **else**20:         No anomaly detected; continue monitoring with KNN at the edge21:     **end if**22:**end while**

### 3.5. Dynamic Workload Management Algorithm

The dynamic workload management algorithm designed and implemented in this paper is one of the core contributions of this research. It has been designed to allocate tasks between the edge and cloud computer in response to network conditions, data volume, and computational demands. The primary objective of the algorithm is to optimize workload distribution to minimize latency *L* and energy consumption *E*, ensuring uninterrupted sensor operations. The overall workload W(t) at time *t* consists of the workloads handled by the edge $W_{edge}\left(t\right)$ and cloud $W_{cloud}\left(t\right)$ expressed in Equation (11).

(11) $$W\left(t\right)=W_{edge}\left(t\right)+W_{cloud}\left(t\right).$$

If $W_{edge}\left(t\right)$ and $W_{cloud}\left(t\right)$ are automatically adjusted to complement each other, the latency will be minimized. The latency functions have been defined as expressed in Equation (12) where $L_{edge}\left(W_{edge}\left(t\right)\right)$ is the latency function for the edge computer and $L_{cloud}\left(W_{cloud}\left(t\right)\right)$ is the latency function for the cloud servers.

(12) $$\min_{W_{edge}\left(t\right),W_{cloud}\left(t\right)}\left(L_{edge}\left(W_{edge}\left(t\right)\right)+L_{cloud}\left(W_{cloud}\left(t\right)\right)\right),$$

However, Equation (12) is subject to constraints on bandwidth *B* and energy *E*. The workload is distributed based on bandwidth availability, which is represented in Equation (13).

(13) $$B\geq \frac{W_{cloud}\left(t\right)}{T_{trans}},$$

In the relation among *B*, $W_{cloud}\left(t\right)$, and $T_{trans}$, $T_{trans}$ represents data transmission time to the cloud. In low-bandwidth conditions, more tasks are processed at the edge to maintain responsiveness. The energy consumption constraint is modeled as Equation (14) where *E* is the energy constraint. The $E_{edge}$ and $E_{cloud}$ represent energy consumption at the edge and cloud, respectively, with $E_{max}$ as the allowable limit.

(14) $$E=E_{edge}\left(W_{edge}\left(t\right)\right)+E_{cloud}\left(W_{cloud}\left(t\right)\right)\leq E_{max},$$

The overall dynamic workload management process follows the process flow presented as Algorithm 2. The incorporation of the dynamic workload management algorithm ensures that the proposed system maintains harmony between the edge computer and the cloud server.
**Algorithm 2** Dynamic Workload Management Algorithm1:**Input:** Sensor data $x_{t}$, bandwidth *B*, max energy $E_{max}$2:**Output:** Optimal $W_{edge}\left(t\right)$ and $W_{cloud}\left(t\right)$ for each cycle3:**while** True **do**         ▹ Continuous monitoring and adjustment4:    Compute $W\left(t\right)=W_{edge}\left(t\right)+W_{cloud}\left(t\right)$5:    Evaluate $L_{edge}\left(W_{edge}\left(t\right)\right)$ and $L_{cloud}\left(W_{cloud}\left(t\right)\right)$6:    **if** Bandwidth *B* is constrained **then**7:        Increase $W_{edge}\left(t\right)$ and reduce $W_{cloud}\left(t\right)$ to minimize transmission8:    **else**9:        Distribute $W_{cloud}\left(t\right)$ to leverage cloud resources10:    **end if**11:    **if** Energy $E_{total}>E_{max}$ **then**12:        Adjust $W_{edge}\left(t\right)$ and $W_{cloud}\left(t\right)$ to reduce power usage13:    **end if**14:**end while**

#### Reproducibility of the Algorithm

To ensure reproducibility, the dynamic workload management algorithm has been enhanced with clear references to previously defined mathematical formulations. The objective function minimizes the sum of latency L(t) and energy consumption E(t), as detailed in Equation (11). Latency L(t) is defined as the sum of edge latency, cloud latency, and data transmission latency (Equation (12)). Similarly, energy consumption E(t) is modeled in Equation (15), capturing contributions from both edge and cloud resources.

The constraints governing bandwidth and energy are critical for managing workload distribution. The bandwidth constraint, expressed in Equation (13), ensures that data transmission to the cloud remains within the available bandwidth B(t). The energy constraint, defined in Equation (14), ensures that the total energy consumption does not exceed the maximum allowable limit $E_{\max}$. Additionally, the workload balancing mechanism ensures that changes in workloads between edge and cloud components are dynamically adjusted to maintain system equilibrium.

By explicitly referencing these equations, the algorithm provides a reproducible framework, allowing researchers and practitioners to replicate and validate the proposed workload management strategy in diverse real-world scenarios.

### 3.6. Resource Optimization Mechanism

One of the contributions of this paper is an innovative resource optimization mechanism, presented as Algorithm 3, which focuses on minimizing both energy consumption $E_{total}$ and bandwidth usage *B* while adhering to latency constraints. The mechanism starts with the principle expressed in Equation (15), where $E_{total}$ is the total energy.

(15) $$E_{total}=E_{edge}+E_{cloud}+E_{trans}$$

In Equation (15), $E_{edge}=W_{edge}\cdot P_{edge}$, $E_{cloud}=W_{cloud}\cdot P_{cloud}$, and $E_{trans}=T_{trans}\cdot P_{trans}$. Here, $T_{trans}$ represents the time required for data transmission, and $P_{trans}$ denotes the power consumption rate for transmission. Bandwidth *B* is determined by data rate *R* and transmission time $T_{trans}$, as defined by Equation (16).

(16) $$B=R\cdot T_{trans}.$$

The optimization objective is updated to include $E_{trans}$ in Equation (17), which is subject to latency $L\leq L_{max}$, where $L=L_{edge}\left(W_{edge}\right)+L_{cloud}\left(W_{cloud}\right)+L_{trans}\left(T_{trans}\right)$.

(17) $$\min_{W_{edge},W_{cloud}}\left(E_{total}+\lambda B\right)$$

**Algorithm 3** Resource Optimization Mechanism
1:**Input:** Workloads $W_{edge},W_{cloud}$; power rates $P_{edge},P_{cloud},P_{trans}$; data rate *R*; latency $L_{max}$2:**while** True **do**           ▹ Iterative loop for continuous optimization3:    Calculate $E_{edge}=W_{edge}\cdot P_{edge}$4:    Calculate $E_{cloud}=W_{cloud}\cdot P_{cloud}$5:    Calculate $E_{trans}=T_{trans}\cdot P_{trans}$ using $T_{trans}=\frac{B}{R}$6:    Compute total energy $E_{total}=E_{edge}+E_{cloud}+E_{trans}$7:    Compute latency $L=L_{edge}\left(W_{edge}\right)+L_{cloud}\left(W_{cloud}\right)+L_{trans}\left(T_{trans}\right)$8:    **if** $L>L_{max}$ or $E_{total}>E_{max}$ **then**9:        Adjust $W_{edge}$ and $W_{cloud}$ to minimize $E_{total}+\lambda B$10:    **end if**11:
**end while**



### 3.7. Security Considerations in Information Processing

The proposed edge-cloud hybrid framework processes and transmits critical sensor data, making it essential to address potential security threats. Security measures are required at every stage: transmission, storage, and processing. This subsection outlines the primary threats and the corresponding mitigation strategies implemented in the framework.

#### 3.7.1. Transmission Security

During data transmission, the system is vulnerable to threats such as eavesdropping, man-in-the-middle attacks, and data tampering. To mitigate these risks, the proposed framework uses end-to-end encryption via Transport Layer Security (TLS). Additionally, secure protocols such as MQTT with SSL/TLS and CoAP with DTLS ensure data integrity and confidentiality during transmission.

#### 3.7.2. Storage Security

Data stored on edge devices and in the cloud are susceptible to unauthorized access and tampering. To address these risks, all stored data are encrypted using Advanced Encryption Standard (AES) with a 256-bit key. Moreover, access controls and multi-factor authentication are implemented to restrict unauthorized access to storage resources.

#### 3.7.3. Processing Security

During processing, security threats include unauthorized code execution and data leakage. To counter these threats, the cloud environment is configured with secure virtual machine isolation, ensuring that no malicious activity can compromise the integrity of the data processing pipeline. Additionally, intrusion detection systems (IDSs) are employed to monitor processing activities for anomalies.

The integration of these security measures ensures that the framework is robust against common security threats, maintaining data integrity, confidentiality, and availability throughout the information processing lifecycle.

## 4. Implementation

**Figure 2:**
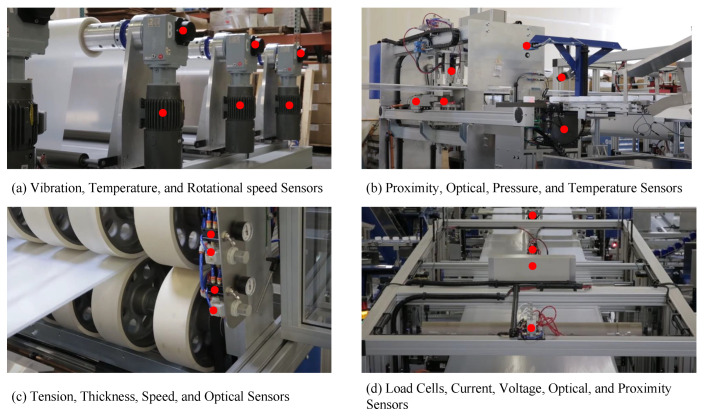
The sensor locations on four different types of machines.

The sensor network used in this study is part of the packaging industry. There are more than 730 sensors in the entire industry. However, only 20 of them were accessible for research and development purposes. These 20 sensors, annotated in Figure 2, are connected to a roller-based winding machine, which is expressed in Figure 2a, an automated packaging machine presented in Figure 2b, a coating machine illustrated in Figure 2c, and conveyor system, which is in Figure 2d. The list of sensors, their specifications, types, and functionalities are listed in Table 9.

### 4.1. KNN Development and Implementation

The KNN applied in this paper to detect the anomaly calculates the Euclidean distance between a new data point and a set of historical data points provided in the dataset defined as $X=\{x_{1},x_{2},\ldots ,x_{n}\}$. Equation (18) has been used to calculate a distance where $x_{i}\in R^{d}$ represents the feature vectors in a *d*-dimensional space.

(18) $$D\left(x_{t},x_{i}\right)=\sqrt{\sum_{j=1}^{d}{\left(x_{t,j}-x_{i,j}\right)}^{2}}.$$

The *k*-nearest neighbors are selected, and the anomaly score $S(x_{t})$ is calculated as the average distance to these *k* neighbors, which is mathematically defined in Equation (19). In this equation, $x_{\left(i\right)}$ represents the *i*-th nearest neighbor in *X*. When $S(x_{t})$ exceeds a predefined threshold $S_{thresh}$, it is considered an anomaly.

(19) $$S\left(x_{t}\right)=\frac{1}{k}\sum_{i=1}^{k}D\left(x_{t},x_{\left(i\right)}\right),$$

**Figure 3:**
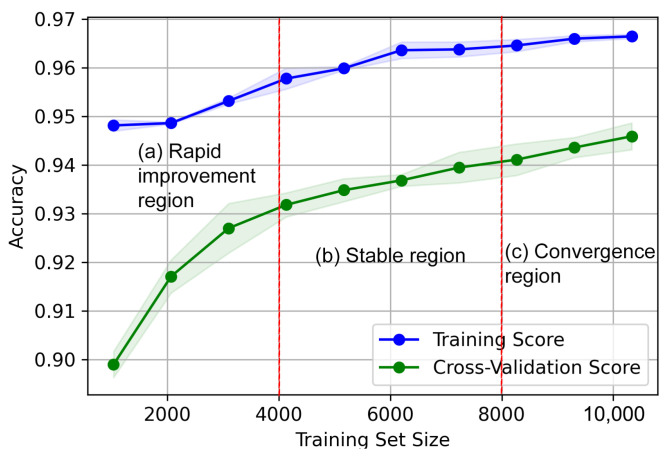
The learning curve analysis of the KNN anomaly detector. The graph is divided into three distinct regions: (a) rapid improvement region, where the accuracy improves significantly with increasing training data; (b) stable region, where the rate of improvement slows down; and (c) convergence region, where accuracy plateaus, indicating diminishing returns with additional data. This division highlights the efficiency of the KNN model in achieving high performance with a moderately sized dataset.

The learning curve analysis presented in Figure 3 demonstrates the learning progress and, at the same time, validation of the learning progress, which justifies that the KNN is properly trained to detect anomalies. It was trained in Google Colab and the trained model was deployed on Raspberry Pi Zero 2 W. The algorithm to detect the anomaly and process the decision has been implemented using Python and the scikit-learn library.

### 4.2. LSTM Network for Failure Prediction in the Cloud

The LSTM network designed and developed in this study processed the sequential sensor data $X_{t}=\left\{x_{t-1},x_{t-2},\ldots ,x_{t-n}\right\}$, where *n* is the sequence length. In this network, each LSTM cell contains three ages, and they are an input gate $i_{t}$, a forget gate $f_{t}$, and an output gate $o_{t}$. The working principles of these gates are governed by Equations (20), (21), and (22), respectively [46].

(20) $$i_{t}=\sigma \left(W_{i}x_{t}+U_{i}h_{t-1}+b_{i}\right)$$

(21) $$f_{t}=\sigma \left(W_{f}x_{t}+U_{f}h_{t-1}+b_{f}\right)$$

(22) $$o_{t}=\sigma \left(W_{o}x_{t}+U_{o}h_{t-1}+b_{o}\right)$$

In these gates, *W*, *U*, and *b* are weight matrices and biases. The σ represents the sigmoid activation function. The cell and hidden states are denoted by $c_{t}$ and $h_{t}$, respectively. The $c_{t}$ and $h_{t}$ have been implemented following their mathematical definitions expressed in Equations (23) and (24) [47].

(23) $$c_{t}=f_{t}\cdot c_{t-1}+i_{t}\cdot \tanh\left(W_{c}x_{t}+U_{c}h_{t-1}+b_{c}\right)$$

(24) $$h_{t}=o_{t}\cdot \tanh\left(c_{t}\right)$$

The LSTM model’s objective is to predict the probability of failure over a future horizon *k* based on past readings. The LSTM network used in this paper has been developed by following the working principles explained earlier. The network architecture has been presented in Table 10. It has been implemented on AWS Lambda using Python and the TensorFlow library.

**Figure 4:**
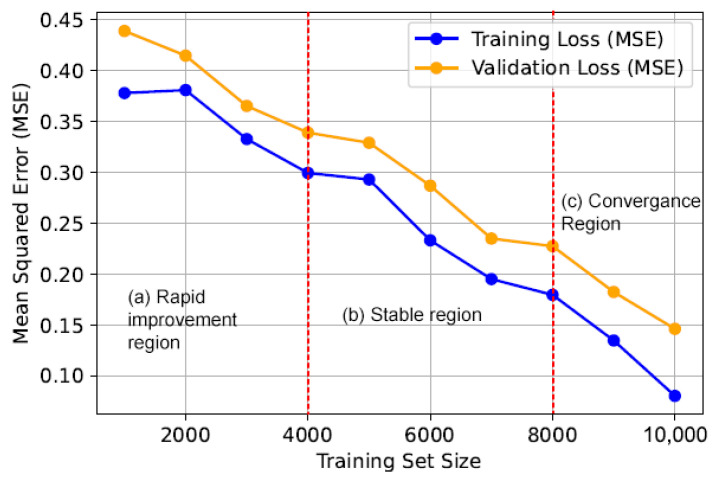
The learning curve analysis of the LSTM failure prediction model. The graph shows three key regions: (a) rapid improvement region, where both training and validation losses decrease substantially; (b) stable region, where the rate of decrease slows, showing consistency; and (c) convergence region, where losses reach minimal levels, indicating a well-trained model with low overfitting risk. These regions emphasize the LSTM’s effective learning and generalization capabilities.

The LSTM network is trained using backpropagation through time (BPTT) to minimize the Mean Squared Error (MSE). Figure 4 illustrates the learning progress of the LSTM network. It shows a consistent decay of MSE of training and validation loss. That means the LSTM network has been trained properly.

### 4.3. Workflow

**Figure 5:**
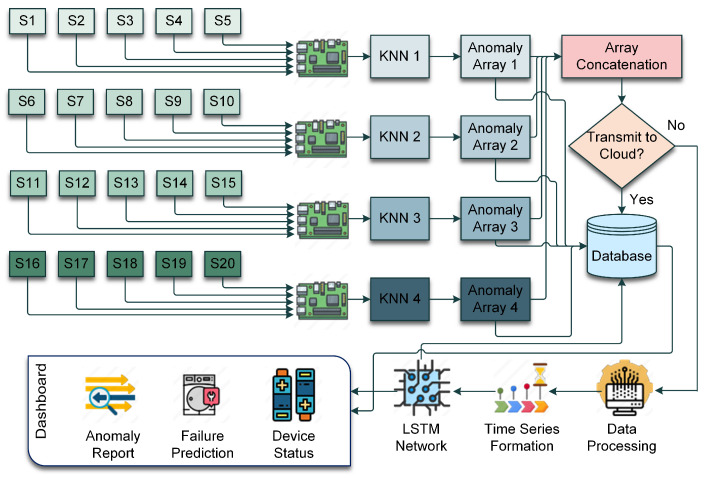
The workflow of the implemented system.

The workflow of the proposed system is illustrated in Figure 5. There are a total of 20 sensors in the experimental environment, which is presented in Figure 2. For ease of operation, five sensors were connected to one Raspberry Pi Zero 2 W. To cover all experimenting sensors, a total of four Raspberry Pi Zero 2 W were used. Each of the edge devices has the trained KNN running. The prediction from the KNN is generated in an array form consisting of zeros and ones where zero represents no anomaly, and one represents an anomaly. After that, these arrays are concatenated. If there are anomalies, only then will the concatenated array be transferred to the cloud. Otherwise, the sensor readings are directly sent to the database. On the cloud, the data are processed for the LSTM network. The sequences are formed as well. After processing, the data are passed to the LSTM network for prediction. The prediction from LSTM, sensor readings, and anomaly status stored in the database is used in the Dashboard, where the anomaly report, failure prediction, and device health are displayed.

## 5. Experimental Results and Performance Analysis

### 5.1. Anomaly Detection at the Edge

**Figure 6:**
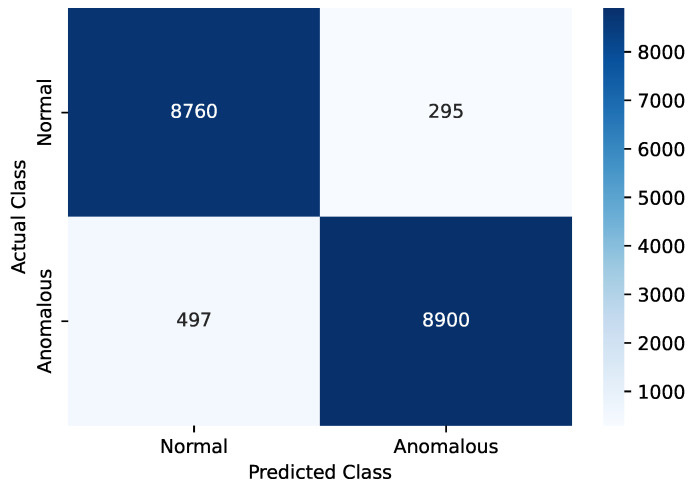
Confusion matrix analysis of the anomaly prediction by KNN.

The KNN model operated from the edge device is responsible for real-time anomaly detection and classifying them into Normal or Anomalous classes. As a classification problem, confusion matrix analysis was used to evaluate performance, as illustrated in Figure 6. In this analysis, 18,452 instances have been used from the test dataset. True Positive (TP), True Negative (TN), False Positive (FP), and False Negative (FN) have been calculated from the confusion matrix. These values are used to calculate the accuracy, precision, recall, and F1-score. The definition of these metrics and their values are listed in Table 11.

**Figure 7:**
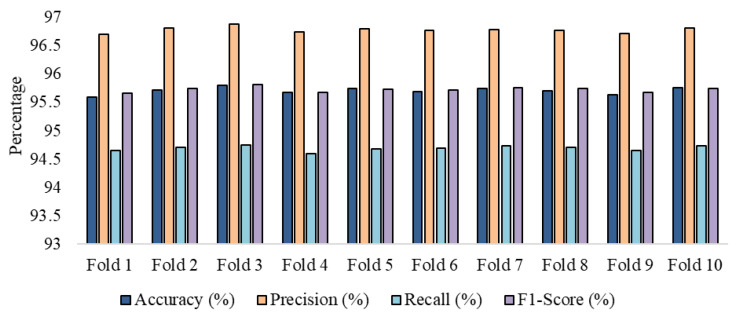
The consistency of performance in all evaluation metrics in k-fold cross-validation.

To further validate the performance of the KNN applied in this paper, k-fold cross-validation has been performed at k=10. The performance in the cross-validation has been illustrated in Figure 7. The graphical demonstration shows that the performance of the anomaly detector at the edge devices is consistent for all classes for every fold. This consistent performance proves that the edge anomaly detector is effective in anomaly detection.

### 5.2. LSTM Failure Prediction at the Cloud

The LSTM network is operated from the AWS Lamda server and designed to predict the probability of failure based on the time-series sensor data. As a regression model, the Mean Absolute Error (MAE) and Root Mean Squared Error (RMSE) were used as evaluation metrics. The definition of MAE and RMSE and their corresponding values are listed in Table 12. The low MAE and RMSE values indicate that the LSTM model provides accurate predictions, enhancing the reliability of failure forecasts in the cloud.

**Figure 8:**
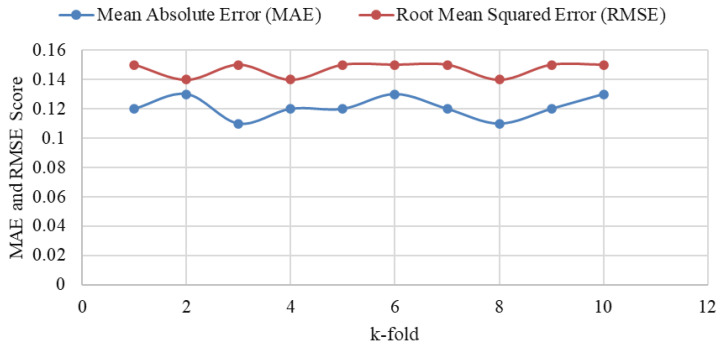
The consistency of MAE and RMSE in the LSTM network in k-fold cross-validation.

A k-fold cross-validation has been performed at k=10 to analyze the consistency of the performance of the failure predictor at the cloud. The performance variations are illustrated in Figure 8. The figure’s MAE and RMSE score range is from 0.02 to 0.16. On this marginal range, it is clearly visible that the LSTM maintains consistent performance in predicting sensor failure.

### 5.3. Latency Performance

The latency plays a crucial role in real-time communication. It has a significant impact on the proposed hybrid edge-cloud framework as well. As a matter of fact, one of the objectives of this paper is to minimize the latency. In the experimental analysis, the system’s latency is assessed by measuring the time taken for anomaly detection at the edge and failure prediction in the cloud. At the same time, we evaluated the end-to-end processing time. The findings from the experimental data are summarized in Table 13. According to the experimental analysis, the latency performance demonstrates the system’s capability for real-time operation. The average end-to-end latency is 500 ms, which is 35% less than the cloud-only solution. This comparison demonstrates that the proposed hybrid framework makes real-time anomaly detection faster.

**Figure 9:**
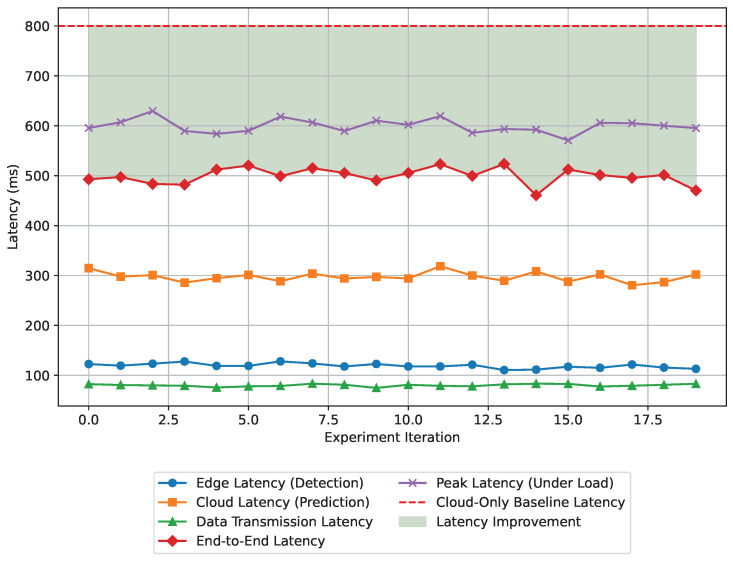
Latency performance of the proposed edge-cloud framework across 20 experiments, showing reductions in end-to-end latency compared to a cloud-only baseline along with other parameters.

The summary presented in Table 13 has been prepared from 20 discrete experiments conducted to evaluate the effect of the proposed hybrid framework on latency. Figure 9 illustrates the latency performance compared against a cloud-only baseline. The latency is reduced significantly because of the efficient workload distribution between the edge and the cloud components. The baseline latency of a cloud-only system is 800 ms, as shown in Figure 9. The green shaded area in the figure highlights the improvement in latency after applying the proposed system. This substantial reduction highlights the advantage of the hybrid model in providing real-time responsiveness, making the proposed system highly suitable for latency-sensitive applications in predictive maintenance.

### 5.4. Energy Consumption Performance

Another objective of the proposed framework is energy efficiency. However, tracking the energy consumption at different end-devices is challenging as it requires additional sensor setup. To avoid the complexities and keep the energy calculation relevant, the energy consumption is measured for anomaly detection at the edge, data transmission, and failure prediction in the cloud, which is not similar to traditional kilowatt/hour calculation. The energy consumption summary is listed in Table 14.

The summary of the energy consumption analysis experiment indicates that the hybrid edge-cloud approach provides significant energy savings. The edge devices are power-efficient computers. As the anomaly is detected at the edge, it saves the energy required to transfer the data and operate the intermediate devices. According to the experimental data, the proposed hybrid framework reduces 28% of energy compared to the cloud-only solution. Another surprising factor discovered during the experiment is that the proposed framework saves an additional 60% of energy savings achieved when operating with edge-only processing during low data flow conditions. The total energy per event remains low enough for scalable deployment in resource-constrained environments.

**Figure 10:**
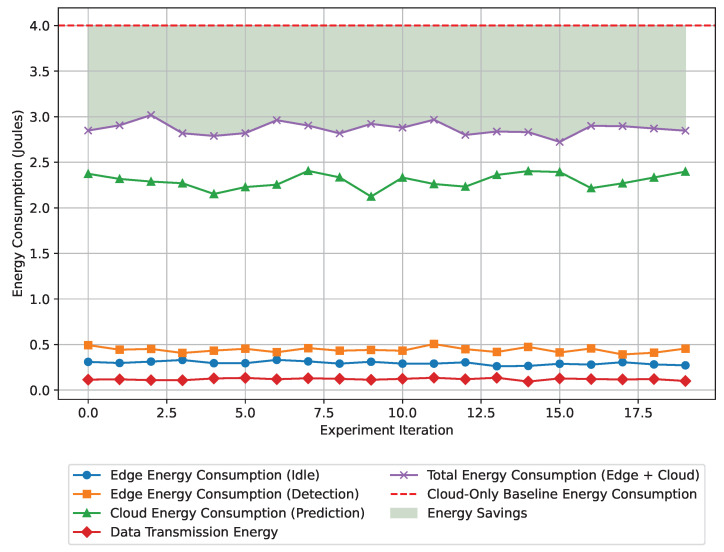
Energy consumption performance of the edge-cloud framework across 20 experiments, showing energy savings compared to a cloud-only baseline.

Figure 10 graphically represents the overall experimental data, the summary of which is in Table 14. It also presents the comparison of the energy savings against the cloud-only system baseline. According to the graphical demonstration, the total energy consumption of the hybrid system consistently falls below the cloud-only baseline, which is highlighted with a green shade. It proves that the proposed system saves substantial energy. The framework’s ability to significantly cut down energy demands demonstrates its suitability for resource-constrained environments, ensuring sustainable operation in real-time predictions.

### 5.5. Bandwidth Usage Performance

Bandwidth is intertwined with the operational cost and feasibility of large-scale sensor networks. Improper bandwidth management can flood the network with traffic, leading to significant transmission delays and impacting real-time communication. During the experiment, the performance of the proposed framework in bandwidth optimization has been evaluated by measuring data transmitted for anomaly reports, regular updates, and peak data flow. The summary of the findings in the experiment is presented in Table 15.

The bandwidth usage analysis shows that each anomaly detection results in 250 KB of data being sent to the cloud for further analysis, while regular updates consume only 50 KB. During peak events, up to 800 KB may be transferred to handle multiple anomalies. The hybrid approach reduces total data transmission by 70% compared to sending all raw data directly to the cloud, with average bandwidth usage per cycle at 150 KB. This represents a bandwidth saving of 60% compared to a cloud-only model, showcasing the benefits of edge processing.

**Figure 11:**
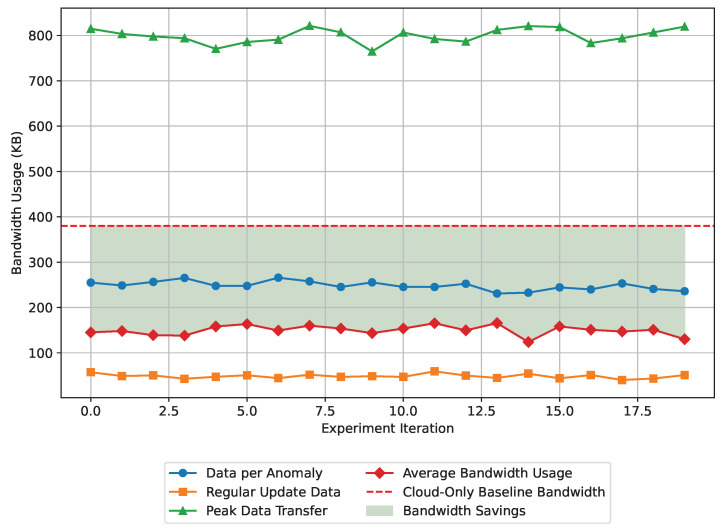
Bandwidth usage performance of the edge-cloud framework across 20 experiments, showing substantial reductions in bandwidth consumption compared to a cloud-only baseline.

Figure 11 illustrates the bandwidth usage performance of the edge-cloud framework across 20 experiments, comparing it to a cloud-only baseline. The green shaded area represents bandwidth savings achieved by limiting data transmission to significant anomaly events rather than continuously sending raw data to the cloud. This approach results in a 60% reduction in average bandwidth usage, demonstrating the efficiency of the framework in conserving network resources for predictive maintenance tasks in real-time.

### 5.6. Comparison with Fog Computing Approaches

Edge computing and fog computing are closely related. Both edge and fog computing are decentralized approaches and contribute to reducing latency [48]. Although these two technologies have some similarities, there are unique characteristics that separate them. Fog computing often involves intermediate nodes positioned closer to data sources than traditional cloud servers, enabling localized data aggregation and processing. It makes the proposed methodology flexible enough to incorporate in the fog computing environment as well. To keep this future scope of research open, a comparison between fog computing and the proposed approach is listed in Table 16. The comparison involves key metrics, including latency, scalability, energy efficiency, and bandwidth usage.

### 5.7. Comparison with State-of-the-Art Workload Management Algorithms

One of the novel contributions of this article is the dynamic workload management algorithm. It is adaptive and actively balances tasks between the edge and cloud resources. The decision is made based on real-time network conditions, data volume, and energy consumption constraints. However, the state-of-the-art workload management algorithms demonstrate promising performance [49]. That is why the performance of the proposed approach has been compared with the existing workload management algorithms. The comparison has been made using four metrics and they are latency, energy efficiency, and resource utilization. Table 17 summarizes the comparison.

The data presented in Table 17 demonstrate that the proposed method is better at workload balancing than other state-of-the-art methods. Because it is dynamic and adaptive, the proposed algorithm performs better and reduces the latency by 35%. At the same time, it improves energy efficiency by 40%. The proposed method shows better performance in scalability and resource utilization as well. This highlights the novelty of the proposed approach, especially in scenarios where computational resources are constrained.

### 5.8. Scalability Considerations for Larger Sensor Networks and Higher Data Velocities

Expansion and sudden increases in data volume are common for sensor networks. Any system developed for the sensor network must be scalable to efficiently perform during these events [55]. The proposed framework has been designed to keep these challenges in consideration. First of all, the system can distribute workloads across a network of devices. Each edge device processes data from a subset of sensors. This is how it ensures that computational demands are shared and no single device is overwhelmed. Secondly, the workload management algorithm dynamically redistributes tasks between edge devices and the cloud based on network conditions and data rates. This adaptability ensures that the system remains efficient even as the scale of the sensor network increases. Lastly, the cloud component of the framework utilizes auto-scaling features to allocate additional computational resources when data velocities are high.

This ensures that the LSTM model continues to provide timely predictions without bottlenecks. An experiment was conducted to analyze the effects of these features. The purpose of this experiment is to find out if these features make the system scalable. The performance-related data obtained from this experiment are listed in Table 18. The results listed in this table demonstrate the framework’s ability to maintain low latency and high efficiency under varying conditions, confirming its applicability in large-scale, real-world deployments.

### 5.9. Comparison with Existing Hybrid Approaches

To further validate the effectiveness of the proposed edge-cloud hybrid framework, a comparative analysis was conducted with existing hybrid approaches available in the literature. The comparison focused on key metrics, including latency, energy efficiency, and bandwidth usage, as summarized in Table 19.

The results indicate that the proposed framework achieves the highest latency reduction (35%), energy efficiency (40%), and bandwidth savings (60%), outperforming other hybrid approaches. Additionally, its scalability is categorized as “Very High” due to the integration of dynamic workload management and cloud auto-scaling mechanisms. These comparisons underscore the novelty and robustness of the proposed system in addressing key challenges in real-time predictive maintenance.

## 6. Limitation and Future Direction

Despite the remarkable achievement of the proposed edge-cloud hybrid framework in latency, energy consumption, and bandwidth optimization, it has several limitations. These limitations are the scope of improvements that provide the future direction of this study. This section highlights these limitations and future directions.

### 6.1. System Downtime

The current framework has no expectation handling mechanism if the cloud server fails to respond. It is not uncommon to experience server downtime in cloud computing [59]. However, this paper considers the ideal case only where the cloud systems ensure uninterrupted service. A backup server during the outage of the main server to support the operation of the hybrid framework is a potential solution to this limitation.

### 6.2. Extendability Issue

The current framework uses a Raspberry Pi Zero 2 W as the edge device. It is a resource-constrained environment with limited capability [60]. Although it is possible to introduce additional features to the existing framework on the cloud server, the scope at the edge node is very limited. From this context, the system suffers from an extendability issue. However, replacing it with a more powerful edge device is an easier solution to this problem. There is a cost-benefit trade-off here, which will be explored in the future scope of this study.

### 6.3. Constraint in Workload Distribution

Although the dynamic workload management algorithm developed and presented in this paper performs properly, because of the limited computational capability at the edge device, the distribution capability suffers from resource constraints. Enhancing the capability of the edge devices makes extending balancing functionality possible. Subsequent versions of this study will explore the potential of this approach.

### 6.4. Sensor Lifespan

One critical issue is the limited lifespan of sensors, especially in harsh environmental conditions, where exposure to dust, humidity, or extreme temperatures can degrade sensor performance over time. Additionally, sensor readings can be influenced by environmental noise, leading to inaccuracies in data collection and processing.

### 6.5. Maintenance Cost

Another significant limitation is the high maintenance cost associated with large-scale sensor networks, as frequent calibration, repair, and replacement are required to ensure consistent performance. For sensors deployed in remote areas, energy efficiency becomes a critical concern, as power supply and data transmission capabilities are often constrained.

Limitations are natural to any system, and the proposed hybrid framework is no exception. However, the way the limitations are handled makes the difference. In this paper, the limitations are considered as the scope of improvements. Further studies are being conducted where these improvements will be presented.

## 7. Keys Aspects and Discussion

The experimental data prove the effectiveness of the proposed edge-cloud hybrid model. This approach is excellent in anomaly detection and sensor failure prediction and significantly reduces operational costs by optimizing resources. This section highlights key aspects of the proposed method.

### 7.1. Industrial Automation and Smart Manufacturing

The edge-cloud hybrid model developed in this paper has the potential to be integrated into industrial automation and smart manufacturing systems. As these systems depend on sensor data to make intelligent decisions, the proposed system is an ideal candidate for ensuring optimal resource usage [61]. Future applications of this model could involve integrating additional data sources like machine telemetry, environmental conditions, and usage history to refine predictive accuracy further and improve maintenance schedules.

### 7.2. Energy Sector and Smart Grids

Another potential field of application of this innovative hybrid model is the energy sector, especially in the smart grids. The modern economy is dependent on the power sector. Even the slightest impact of infrastructural or technical damage on the power sector causing power supply disruption can seriously impact numerous businesses [62]. That is why predictive maintenance is crucial in this sector. The successful integration of the proposed edge cloud can significantly enhance the reliability of power-generating systems, including wind turbines, solar panels, hydro turbines, and other sources managed by the smart grids.

### 7.3. Environmental Monitoring and Agriculture

The proposed edge-cloud hybrid model has the potential to ensure sustainability in smart environmental monitoring and agriculture. These two sectors are heavily dependent on sensors, which are often damaged because of the harsh environmental effects [63]. This is one of the reasons that significantly impacts the sustainability of this field. However, applying the proposed edge-cloud hybrid model can change and enhance sustainability by predicting sensor failure with minimal computational resources.

The innovative edge-cloud hybrid model supported by the KNN at the edge and LSTM network at the cloud is a technology with the potential of being integrated with a sensor network wherever predictive maintenance is required. It has unlocked a wide range of opportunities, which will be explored in the future scope of this study.

## 8. Conclusions

This paper presented a novel edge-cloud hybrid framework designed to address key challenges in predictive maintenance for sensor networks. By strategically combining the low-latency capabilities of edge computing with the extensive computational power of cloud servers, this framework achieves significant improvements in latency, energy consumption, and bandwidth usage. The KNN-based anomaly detection model, deployed at the edge, enables real-time anomaly detection, reducing the frequency of data transmissions to the cloud and minimizing latency. The cloud-hosted LSTM network, in turn, provides accurate long-term failure predictions, allowing for timely maintenance scheduling and operational optimization.

The experimental results demonstrate that this hybrid approach provides a 35% reduction in latency and 28% energy savings compared to a cloud-only model. Additionally, the bandwidth usage is reduced by 60%, underscoring the efficiency of processing data locally at the edge before transmission. These results highlight the framework’s applicability in real-time predictive maintenance, particularly in resource-constrained environments. The proposed edge-cloud hybrid framework was also compared with existing hybrid approaches from the literature. As shown in Table 19, it outperformed other methods in latency reduction, energy efficiency, and bandwidth savings while demonstrating superior scalability. These findings further establish the framework’s competitive advantage and its potential for practical applications in resource-constrained environments.

In conclusion, this hybrid edge-cloud approach provides a promising solution for real-time predictive maintenance in sensor networks. The balanced use of edge and cloud resources demonstrated in this study serves as a foundation for further research into scalable, efficient, and cost-effective frameworks for the next generation of smart industrial applications.

## Figures and Tables

**Table 1:** Sample of dataset with key features.

Timestamp	$x_{t1}$	$x_{t2}$	$x_{t3}$	$x_{t4}$	$x_{t5}$	$x_{t6}$	$y_{t}$	$z_{t}$
2024-11-08 00:00:00	0.75	0.40	0.65	0.80	0.33	0.55	Normal	0
2024-11-08 00:01:00	0.77	0.38	0.68	0.82	0.32	0.56	Normal	0
2024-11-08 00:02:00	0.72	0.41	0.63	0.79	0.35	0.57	Anomalous	1
2024-11-08 00:03:00	0.76	0.39	0.66	0.81	0.34	0.58	Normal	0

**Table 2:** Comparison of edge devices for real-time anomaly detection.

Device	Manufacturer	CPU	RAM	Power Consumption	Cost
Raspberry Pi Zero 2 W	Raspberry Pi Foundation, Cambridge, UK	Quad-core 1.0 GHz	512 MB	Low	Low
NVIDIA Jetson Nano	NVIDIA Corporation, Santa Clara, CA, USA	Quad-core 1.43 GHz	4 GB	Moderate	High
Coral Edge TPU	Google, Mountain View, CA, USA	Dual-core Cortex-A53	1 GB	Low	Moderate
Arduino Nano 33 IoT	Arduino AG, Somerville, MA, USA	ARM Cortex-M0+	32 KB	Very Low	Very Low

**Table 3:** Comparison of AI models for edge-based anomaly detection.

Model	Computational Complexity	Memory Usage	Accuracy
K-Nearest Neighbors (KNNs)	Low	Low	Moderate
Decision Tree	Moderate	Moderate	High
Support Vector Machine (SVM)	High	High	High
Naive Bayes	Low	Very Low	Low

**Table 4:** Comparison of cloud computing services for deep learning.

Cloud Service	Scalability	Latency	Cost
AWS Lambda	High	Low	High
Google Cloud Functions	High	Moderate	Moderate
Microsoft Azure Functions	Moderate	Low	Moderate
IBM Cloud Functions	Moderate	Moderate	Low

**Table 5:** Comparison of deep learning models for cloud-based failure prediction.

Model	Suitability for Time-Series	Computational Complexity	Accuracy
LSTM	High	High	High
GRU	Moderate	Moderate	Moderate
CNN	Low	High	Moderate
RNN	High	Moderate	High

**Table 6:** Comparison of metrics for the KNN method.

Metric	Classification Accuracy (%)	Computational Complexity
Euclidean Distance	95.71	Low
Manhattan Distance	95.72	Moderate
Minkowski Distance (*p* = 3)	95.70	High

**Table 7:** Impact of window width on KNN Performance.

Window Width (Time Steps)	Classification Accuracy (%)	Processing Time (ms)
10	93.41	100
20	94.86	120
30	95.71	140
40	95.30	160
50	94.90	190

**Table 8:** Comparison of data transfer protocols and their limitations [45].

Protocol	Latency	Bandwidth Efficiency	Security Features	Limitations
MQTT	Low	High	Basic encryption (TLS)	Limited Quality of Service (QoS) in high-traffic networks
HTTP	Moderate	Moderate	Robust (HTTPS)	High overhead for frequent small messages
WebSocket	Low	Moderate	Basic encryption (TLS)	Susceptible to connection interruptions
CoAP	Very Low	High	Basic encryption (DTLS)	Limited scalability for large deployments

**Table 9:** Specifications and functionalities of sensors used in the study.

Sensor	Specifications	Type	Functionality
Vibration Sensor	Range: ±25 g, Frequency: 10 Hz to 1 kHz, Sensitivity: 100 mV/g	Accelerometer	Monitors vibration levels to detect mechanical faults
Temperature Sensor	Range: −50 °C to +200 °C, Accuracy: ±0.5 °C	Thermocouple, RTD, Thermistor	Measures temperature to identify overheating and lubrication issues
Rotational Speed Sensor	Range: 0 to 10,000 RPM, Accuracy: ±1%	Optical, Magnetic	Monitors rotational speed to ensure safe operation limits
Proximity Sensor	Detection Range: Up to 30 mm	Inductive, Capacitive, Ultrasonic	Detects object presence/ absence for positioning and safety
Optical Sensor	Range: 10 mm to 2 m, Resolution: 0.01 mm	Photodetector, Infrared	Detects objects, measures distances, verifies positioning
Pressure Sensor	Range: 0–500 psi, Accuracy: ±0.25% FS	Piezoresistive, Capacitive	Measures pressure for hydraulic/ pneumatic systems
Tension Sensor	Range: 0–1000 N, Accuracy: ±0.5% FS	Strain Gauge	Measures material tension to maintain quality
Thickness Sensor	Range: 0–100 mm, Resolution: 0.01 mm	Laser-based, Ultrasonic	Measures thickness in processes like rolling and coating
Speed Sensor	Same as Rotational Speed Sensor	Optical, Magnetic	Measures speed of parts in assembly lines for synchronization
Load Cell	Range: 0–500 kg, Accuracy: ±0.1% FS	Strain Gauge, Piezoelectric	Measures force/load, commonly for weight measurements
Current Sensor	Range: 0–100 A, Accuracy: ±1%	Hall Effect, Shunt Resistor	Monitors electrical current to detect overloads or malfunctions
Voltage Sensor	Range: 0–1000 V, Accuracy: ±0.5%	Potential Transformer, Hall Effect	Measures voltage levels for safe operation

**Table 10:** Characteristics and features of the LSTM network for failure prediction.

Feature	Description
Input Sequence Length	30 time steps (sensor data sequences)
Input Dimensions	20 (number of sensor features per time step)
LSTM Layers	2 layers
Units per LSTM Layer	50 units
Activation Function	Sigmoid for gates, Tanh for cell states
Output Layer	Fully connected layer for regression output
Loss Function	Mean Squared Error (MSE)
Optimizer	ADAM
Learning Rate	0.001 (initial learning rate)
Batch Size	32
Epochs	100
Dropout Rate	0.2 (for regularization to prevent overfitting)
Training Data	Historical time-series sensor data
Deployment Environment	AWS Lambda
Scalability	Automatically scales with AWS Lambda for large datasets
Prediction Target	Failure probability within future horizon

**Table 11:** Performance metrics for KNN anomaly detection at the edge.

Metric	Formula	Value
Accuracy	$\frac{TP+TN}{TP+TN+FP+FN}$	95.71%
Precision	$\frac{TP}{TP+FP}$	96.79%
Recall	$\frac{TP}{TP+FN}$	94.71%
F1-Score	$2\cdot \frac{\mathrm{Precision}\times \mathrm{Recall}}{\mathrm{Precision}+\mathrm{Recall}}$	95.74%

**Table 12:** Performance metrics for LSTM failure prediction in the cloud.

Metric	Formula	Value
Mean Absolute Error (MAE)	$\frac{1}{N}\sum_{i=1}^{N}\left|y_{actual}\left(i\right)-y_{pred}\left(i\right)\right|$	0.12
Root Mean Squared Error (RMSE)	$\sqrt{\frac{1}{N}\sum_{i=1}^{N}{\left(y_{actual}\left(i\right)-y_{pred}\left(i\right)\right)}^{2}}$	0.15

**Table 13:** Latency performance of the edge-cloud framework.

Metric	Description	Value (ms)
Edge Latency (Detection)	Average time for KNN anomaly detection	120
Cloud Latency (Prediction)	Average time for LSTM failure prediction	300
Data Transmission Latency	Average time to transmit data from edge to cloud	80
End-to-End Latency	Total time for edge detection and cloud prediction	500
Peak Latency (Under Load)	Max observed latency during high data flow	600
Latency Reduction (vs. Cloud-only)	Improvement over a cloud-only solution	35%

**Table 14:** Energy consumption performance of the edge-cloud framework.

Metric	Description	Value (J)
Edge Energy Consumption (Idle)	Baseline energy consumption in idle state	0.30
Edge Energy Consumption (Detection)	Energy per anomaly detection event (KNN)	0.45
Cloud Energy Consumption (Prediction)	Energy per failure prediction event (LSTM)	2.30
Data Transmission Energy	Energy for transmitting data to cloud per event	0.12
Total Energy Consumption (Edge + Cloud)	Combined energy for detection, transmission, and prediction	2.87
Energy Reduction (vs. Cloud-only)	Energy savings compared to cloud-only processing	28%
Energy Efficiency (Edge-only Processing)	Energy saved when processing anomalies at edge only	60%

**Table 15:** Bandwidth usage performance of the edge-cloud framework.

Metric	Description	Value (KB)
Data per Anomaly	Average data sent to cloud per anomaly detected	250
Regular Update Data	Data sent periodically to update cloud without anomalies	50
Peak Data Transfer	Data transferred during peak anomaly events	800
Data Reduction (vs. Raw Transmission)	Reduction from transmitting only anomalies	70%
Average Bandwidth Usage	Average bandwidth used per operation cycle	150
Bandwidth Savings (vs. Cloud-only)	Savings from processing data at the edge	60%

**Table 16:** Comparison of proposed hybrid framework with fog computing approaches.

Metric	Fog Computing	Proposed Hybrid Framework
Latency	Very Low (localized processing)	Low (edge anomaly detection, cloud prediction)
Scalability	Limited by fog node resources	High (scalable cloud resources)
Energy Efficiency	Moderate (fog nodes consume power)	High (edge devices are energy- efficient)
Bandwidth Usage	Low (localized processing reduces data transfer)	Low (edge filtering reduces data transfer)
Computational Complexity	High (fog nodes handle extensive tasks)	Balanced (edge for lightweight tasks, cloud for heavy analytics)

**Table 17:** Comparison of workload management algorithms.

Algorithm	Latency Reduction (%)	Energy Efficiency (%)	Resource Utilization (%)	Scalability
Static Partitioning [50]	15	20	65	Moderate
Heuristic-Based Approach [51]	20	25	70	High
AI-Driven Dynamic [52]	30	35	85	High
Multi-Agent Learning (Malcolm) [53]	28	34	80	High
SDN-Based Hybrid Load Balancing [54]	25	30	75	High
Proposed Algorithm	35	40	90	Very High

**Table 18:** Scalability analysis of the proposed framework.

Scenario	Sensor Count	Data Velocity (MB/s)	Latency (ms)	Energy Efficiency (%)
Small Network	20	1	500	90
Medium Network	100	10	600	85
Large Network	500	50	700	80
Very Large Network	1000	100	800	75

**Table 19:** Comparison of hybrid frameworks.

Approach	Latency Reduction (%)	Energy Efficiency (%)	Bandwidth Savings (%)	Scalability
Dynamic Deployment Framework [56]	20	25	50	Moderate
Jay: Offloading Framework [57]	30	35	55	High
Hybrid Federated Edge Learning [58]	25	30	60	High
Proposed Edge-Cloud Framework	35	40	60	Very High

## Data Availability

The data utilized in this experiment are derived from real-world industrial sources and contain confidential information. These data are available for research purposes upon reasonable request, provided that the recipient agrees not to use them for commercial purposes and not to share them in any form. Upon acceptance of these terms, access to the data will be granted by the corresponding authors.

## Abbreviations

The following abbreviations are used in this manuscript:

AI	Artificial Intelligence
DL	Deep Learning
ML	Machine Learning
KNN	K-Nearest Neighbors
LSTM	Long Short-Term Memory
MAE	Mean Absolute Error
MSE	Mean Squared Error
RMSE	Root Mean Squared Error
IoT	Internet of Things
3Vs	Volume, Velocity, Variety
AWS	Amazon Web Services
TP	True Positive
TN	True Negative
FP	False Positive
FN	False Negative
